# Exploring Youths’ Offers to Use E-Cigarettes in Rural Hawai‘i: A Test Development and Validation Study

**DOI:** 10.3390/ijerph21111427

**Published:** 2024-10-28

**Authors:** Scott K. Okamoto, Andrew M. Subica, Kelsie H. Okamura, Katlyn J. An, Sarah D. Song, Paula Angela Saladino, Adabelle B. Carson, Zarek K. Kon, Sarah Momilani Marshall, Steven Keone Chin, Joseph Keawe‘aimoku Kaholokula, Ian Pagano, Pallav Pokhrel

**Affiliations:** 1 Population Sciences in the Pacific Program, University of Hawai‘i Cancer Center, 701 Ilalo St., Honolulu, HI 96813, USA; ankatlyn@hawaii.edu (K.J.A.); sarahds@hawaii.edu (S.D.S.); paula20@hawaii.edu (P.A.S.); abcarson@hawaii.edu (A.B.C.); zarekkon@hawaii.edu (Z.K.K.); schin@hawaii.edu (S.K.C.); pagano@hawaii.edu (I.P.); ppokhrel@cc.hawaii.edu (P.P.); 2 School of Medicine, University of California Riverside, 900 University Ave., Riverside, CA 92521, USA; andrew.subica@ucr.edu; 3 The Baker Center for Children and Families, 53 Parker Hill Ave., Boston, MA 02120, USA; kokamura@jbcc.harvard.edu; 4 Thompson School of Social Work & Public Health, University of Hawai‘i at Mānoa, Gartley Hall, 2430 Campus Road, Honolulu, HI 96822, USA; smm93@hawaii.edu; 5 John A. Burns School of Medicine, University of Hawai‘i at Mānoa, 677 Ala Moana Blvd. Suite 1016B, Honolulu, HI 96813, USA; kaholoku@hawaii.edu

**Keywords:** Native Hawaiian, Pacific Islander, youth, e-cigarette, test development

## Abstract

The purpose of this study is to describe the development and initial validation of a survey focused on problematic situations involving e-cigarette use by rural Native Hawaiian and Pacific Islander (NHPI) youths. A 5-phase approach to test development and validation was used. In Phase 1 (Item Generation), survey items were created from a series of focus groups with middle school youths on Hawai‘i Island (*N* = 69). In Phase 2 (Item Refinement and Selection), situational items were reduced to 40 e-cigarette offer situations that were selected for inclusion in the survey. In Phase 3 (Item Reduction), items were administered to 257 youths from 11 middle, intermediate, or multi-level public or public-charter schools on Hawai‘i Island. Exploratory factor analysis indicated the presence of three factors accounting for 50% of the variance: E-Cigarette Offers from Friends (24%), E-Cigarette Offers from Non-Friends (16%), and Coercive Pressure to Use E-Cigarettes (10%). Hypothesized relationships between offer situations and e-cigarette use were partially confirmed, supporting the construct validity of the survey. This survey helps to fill the scientific and practice gap in measuring ecodevelopmental risk and protection for e-cigarette use and has implications for e-cigarette use prevention with rural, NHPI, and/or Indigenous youth populations.

## 1. Introduction

Over the past decade, an exponential growth of youth e-cigarette use has emerged as a major health concern [1]. E-cigarettes have consistently remained the most commonly used tobacco product among youths in the United States, with 10% of high school students and 4.6% of middle school students recently reporting use of these devices within the past 30 days [2]. Recent data have also indicated that youths’ e-cigarette use is directly associated with their development and/or exacerbation of severe respiratory disorders, such as COVID-19 [3] and asthma [4,5]. Additionally, a recent scoping review found that youth e-cigarette use is associated with chronic obstructive pulmonary disease [5]. Research that informs the antecedents and/or contributors to youth e-cigarette use is a necessary step toward understanding and preventing the use of these devices and their related adverse health outcomes, particularly within diverse and hard-to-reach populations.

The purpose of this study is to examine socially and developmentally relevant offers to use e-cigarettes experienced by a predominantly Native Hawaiian and Pacific Islander (NHPI) sample of early adolescents in rural Hawai‘i. Recent epidemiological data have found that 18% of all middle school youths in the state of Hawai‘i currently use an electronic vapor product, ranking first nationally among all states collecting data on middle school youths. Of the youths who report current use, 30% of them are of NHPI ancestry, representing the highest percentage of e-cigarette users among major ethnic groups in Hawai‘i. Guided by ecodevelopmental theory [6], we used a 5-phase test development and validation procedure to develop an initial inventory of situations in the home, school, and community involving youths’ offers to use e-cigarettes. Our study contributes to the understanding and measurement of the social and contextual contributors to e-cigarette use initiation for rural NHPI youths. As a result, it will contribute to emerging e-cigarette use prevention efforts for Pacific and/or Indigenous youth populations.

## 2. Literature Review

### 2.1. NHPI Youths and E-Cigarette Use

While there have been several studies examining NHPI youth substance use rates and related adverse outcomes [7,8,9,10], the majority of these studies predated the emergence of electronic vapor products and, therefore, provide limited insight into the contexts and contributors to e-cigarette use for these youths. Further, the majority of the published literature on e-cigarette use has focused on NHPI young adults, examining their rates and correlations of use. For example, a recent systematic literature review of NHPI tobacco product use found that social influence and favorable expectancies toward e-cigarette use were positively related to NHPI young adults’ use of these devices [11]. Additionally, a study of NHPI young adults found that their e-cigarette use exceeded that of other at-risk racial groups and called for more research to understand the risk and protective factors for this population [12].

Research focused specifically on e-cigarette use of NHPI early adolescents is beginning to emerge. A recent study examined the strategies that NHPI youths used to deal with the widespread and persistent social demands to use e-cigarettes in rural Hawai‘i [13]. Four strategies were primarily used to refuse offers to use e-cigarettes by these youths—refuse (saying “no”), explain (providing an explanation for refusal), avoid (staying away from locations or situations where e-cigarette offers occur), and leave (walking away from a situation where e-cigarettes were being offered). While the youths’ strategies identified in the Okamoto et al. (2024) study paralleled those from prior research broadly focused on substance use [14], narratives from the former study suggested a diminished efficacy in their use within e-cigarette offer situations. This was evidenced by the frequent need to repeat or combine strategies to deal with multiple, and sometimes coercive, offers to use electronic vapor devices in their homes, schools, and communities. 

### 2.2. Ecodevelopmental Theory

Ecodevelopmental theory emphasizes that the development and behavior of an individual is shaped by their relationship to their environment [6,15,16]. Within the present study, the theory can be used as a framework for understanding how offering e-cigarettes can influence their use by NHPI youths in rural communities. Specifically, NHPI youths’ e-cigarette use can be understood as the result of explicit or implicit offers to use these devices that occur within nested systems. *Microsystems* are structures in which the youths participate, such as the family, school, and peer groups. Ecodevelopmental theory particularly focuses on the role of the family system in the socialization of youths and the development or prevention of problem behaviors [15]. *Mesosystems* reflect the relationships between microsystems (e.g., parent–peer or parent–school interactions), which exert an indirect influence on youths. *Macrosystems* reflect broad social forces and structures that influence youths, including their cultural context. In the present study, ecodevelopmental theory is operationalized within the e-cigarette offer situations identified by the NHPI youths. These situations reflect the specific social, cultural, and developmental contexts of the families, schools, and communities in rural Hawai‘i, as well as the unique demands faced by these youths to use e-cigarettes.

Ecodevelopmental theory has been used in past substance use prevention research with diverse youth populations, including American Indian [17] and Latino [15,18] youths. For example, Marsiglia and colleagues used the theory to explain why culturally grounded substance use prevention did not exert stronger preventive effects for elementary versus middle school-aged Latino youths [18]. They concluded that the cultural and familial context emphasized in the theory exerted a more substantial protective effect on younger children. As they approached adolescence, these sociocultural influences eroded, and the peer context began to exert a stronger influence, requiring substance use prevention to counteract their influences.

### 2.3. Relevance of the Study

This study contributes to the measurement literature focused on e-cigarette-related constructs. A review of this literature found 23 studies identifying 22 different instruments, with the majority focused on measuring internalized constructs, such as beliefs, perceptions, and/or motives for e-cigarette use [19]. Park and colleagues noted a gap in the measurement literature focused on diverse and youth populations and the lack of studies focused on the social or cultural contexts of e-cigarette use. The present study fills this gap because it elucidates the unique social and cultural demands faced by NHPI youths to accept offers to use e-cigarettes. The findings build upon recent research highlighting the salience of social influence as a reason to use e-cigarettes [20] and inform a broader body of literature focused on the social and cultural contexts of e-cigarette use for diverse youth populations. Finally, the present study addresses the social and contextual contributors to e-cigarette use for an underserved population with substantial tobacco product use disparities. It provides an initial step toward the measurement of these contributors and an instrument for the sociocultural assessment of e-cigarette use and/or the evaluation of e-cigarette interventions for rural NHPI youth populations. 

## 3. Method

Similar to prior research [21], this study utilized a 5-phase, culturally grounded approach to test development and validation with rural NHPI youths. Qualitative methods were used in phases 1 and 2 of the research (item generation and item refinement and selection) to create survey items, while quantitative methods were used in phases 3–5 (item reduction, reliability, and validity) to validate and evaluate these items within public and public-charter middle, intermediate, and multi-level schools on Hawai‘i Island. Hawai‘i Island is one of four main islands in the state of Hawai‘i, with 98.7% of its land area designated as rural [22]. All research procedures were approved by the Institutional Review Boards at the University of Hawai‘i at Mānoa and the State of Hawai‘i Department of Education, and all research participants provided both active parental informed consent and student assent for their participation in all phases of this study.

### 3.1. Phase 1: Item Generation

The goal of this phase was to generate a large, representative pool of potential survey items related to youths’ offers to use e-cigarettes. The items were created based on narrative descriptions from a predominantly rural NHPI youth sample (*N* = 69) participating in one of seventeen gender-specific (i.e., all-male or all-female) focus groups. Forty-eight, forty-two, and nine percent of the sample identified as female, male, and non-binary, respectively. Non-binary youths chose to participate in the female focus groups. These focus groups were conducted across eight low-income, geographically dispersed middle, intermediate, and multi-level public or public-charter schools on Hawai‘i Island. These schools represented each of the three school complex areas in the Hawai‘i Department of Education on Hawai‘i Island, reflecting approximately 50% of all public and public charter schools on the island. The sample was recruited in collaboration with school-based research liaisons, who were faculty or staff members in the schools (e.g., school counselors and health teachers) who assisted the research team in recruiting focus group participants. Liaisons focused on recruiting primarily NHPI youths in their schools who were willing to discuss e-cigarette offers and use. Participants were recruited across three demographic groups—(1) e-cigarette users (38%), (2) e-cigarette contemplators (i.e., youths who were considering trying e-cigarettes; 21%), and (3) non-e-cigarette users (41%). Youth participants (M_age_ = 12.5 years) described situations where e-cigarettes were offered to them or someone they knew and the challenges they faced in dealing with these offers in their homes, schools, and communities. Selected questions from the semi-structured interview schedule used to guide the discussions are included in Table 1. Focus groups took approximately 45 min to complete, and participants received a $5.00 gift card to a local store and snacks as compensation for their time and effort. The focus group data were transcribed verbatim, checked for accuracy, and systematically coded to identify statements or descriptions of specific situations where e-cigarettes were offered to youths. This process yielded a total of 78 potential survey items from the focus group transcripts. As a result of this process, the items reflected the specific ecodevelopmental contexts of the youth participants, including offers from friends, cousins, parents, and aunts/uncles in a variety of rural settings and locations.

### 3.2. Phase 2: Item Refinement and Selection

The goals of this phase were to reduce the number of items to be included in the survey and to establish two measures for each item: (1) the *frequency* in which the youths were exposed to each situation and (2) the perceived *difficulty* that youths had in refusing e-cigarettes in each situation. The total number of situations was reduced by eliminating redundant, non-representative, and vague items and combining similar items. To control bias, item refinement, and selection were first conducted individually by research team members, followed by a team validation process where members justified items for refinement, inclusion, or exclusion from the survey. Items that were too specific or appeared random in nature were eliminated. For example, one eliminated item focused on how a classmate who wanted to “get rid of his vape to avoid getting caught” would put it in their hand and then offer to shake your hand to pass off the e-cigarette to you during class. This item was deemed too behaviorally specific and overlapped with another item focused on sharing an electronic vaping device in class; therefore, it was eliminated. Items that were mentioned within two or more focus groups were prioritized for inclusion in the final item list (*n* = 23 items). Additional items were included based on the team validation process (*n* = 17).

This process produced a final list of 40 items focused on youths’ offers to use e-cigarettes (see Table 2 for a full list of the e-cigarette items). Forty percent of the items described a friend as a primary e-cigarette offeror, followed by a peer (30%), classmate (10%), sibling (7%), cousin (5%), parent/aunt/uncle (5%), and boy/girlfriend (3%). Thirty-seven percent of the items described an e-cigarette offer in the school setting, followed by an unspecified location (35%), a home setting (18%), and a community setting (e.g., park; 10%). Students completing the survey were instructed to use two Likert scales to respond to each item—one rating the lifetime frequency of experiencing the situation, ranging from 1 (“Never”) to 5 (“More than 10 times”), and the other rating the perceived difficulty in refusing e-cigarettes in the situation, ranging from 1 (“Very easy”) to 5 (“Very difficult”).

### 3.3. Phase 3: Item Reduction

*Participants*. Two hundred and fifty-seven youths from 11 different middle, intermediate, or multi-level schools on Hawai‘i Island completed the Phase 2 survey. These youths were sampled from the same regions as the youths participating in Phase 1 of the study. Participants in Phase 3 may have also participated in Phase 1 of the study, although this was not tracked across schools. Similar to Phase 1, youth participants were recruited in collaboration with school-based research liaisons, who assisted the research team with distributing and collecting parental consent forms, secured space in the schools for in-person online or hard-copy survey administration, and encouraged youths to participate in the study. The demographics of the sample are presented in Table 3.

*Procedures*. Surveys were administered in the classroom setting by members of the research team. Youth participants took the survey online (*n* = 226) or by hard copy (*n* = 31). The online version of the survey was delivered through Research Electronic Data Capture (REDCap) hosted at the University of Hawai‘i at Mānoa. REDCap is a secure web application for building and managing online surveys and databases [23]. Each student was given an ID number upon verification of signed parental consent. This unique number allowed youths to access the student assent form and survey on their laptop computers. To promote comprehension of the survey items, a member of the research team read each question aloud and encouraged the students to follow along. The survey included demographic questions (e.g., age, gender, ethnicity), risk and protective factor questions (e.g., substance use questions, such as past 30-day vaping of flavors/e-liquid, marijuana vaping, and other substance use), and the frequency and difficulty ratings of problematic situations involving offers to use e-cigarettes. Surveys took approximately 40 min to complete. Students received a $5.00 gift card to a local store as compensation for their time and effort. 

*Exploratory Factor Analysis*. Exploratory factor analysis was conducted on the frequency scale of the survey. The procedure was not conducted on the difficulty scale, as past substance use research with NHPI youths found a lack of factor interpretability based on similar difficulty assessments [21]. A correlation matrix of questionnaire items for the frequency scale was completed to examine relationships among items. Principal axis factoring with Promax rotation was performed on the full sample in order to identify and validate latent constructs. A common factor model rather than a principal components analysis was selected to control for the influences of specific variance and error variance on the factor structure. An oblique versus orthogonal rotation was selected based on the predicted correlation between potential factors.

The number of factors was determined using a scree plot. Scree plots consist of the magnitude of eigenvalues as a function of their ordinal position [24], and using them to determine the number of retained factors in exploratory factor analysis is preferred over other methods [25]. Because the primary criticism of the use of the scree plot is its reliance on subjectivity [26], several plausible factor solutions were considered and analyzed based on the natural bend and curve flattening in the screen test in order to determine the most interpretable solution. We applied a more conservative variation of the 0.40–0.30–0.20 rule to determine which items to retain within factors [27]. For this study, items were retained if they (a) loaded onto their primary factor at or above 0.55, (b) loaded onto alternative factors below 0.30, and (c) demonstrated a difference of 0.20 or more between their primary and alternative factor loadings. Cutoffs at or above 0.55 fall within the “good” to “excellent” range [28]. In order to control sample bias, we employed pairwise deletion of missing survey data.

### 3.4. Phase 4: Reliability

Internal consistency was assessed with Cronbach’s alphas for each of the subscales derived from the factor analysis.

### 3.5. Phase 5: Validity

In order to test the construct validity of the measure, three hypotheses were proposed and tested.

*Hypothesis 1.* Higher grade levels of youth participants will be associated with increased exposure to e-cigarette-related offer situations. This hypothesis is based on U.S. data collected since 2015 that have demonstrated increases in e-cigarette use as youths matriculate from middle to high school [29].

*Hypothesis 2.* Youths’ increased exposure to e-cigarette-related offer situations will be associated with higher levels of their e-cigarette use. This hypothesis is based on research with rural Hawaiian youths, which found that exposure to offers to use alcohol, tobacco, and other drugs (ATOD) from peers and family members was predictive of ATOD use [7].

*Hypothesis 3.* Youths’ increased difficulty in refusing e-cigarettes in offer situations will be associated with higher levels of e-cigarette use. This hypothesis is based on research that found that early adolescent youths who were less familiar with effective drug resistance skills or were less confident in using them were more prone to ATOD use [30].

## 4. Results

### 4.1. Phase 3: Item Reduction

*Descriptive Statistics*. For the frequency scale, mean scores for the 40 items ranged from 1.07 (items 2, 10, and 13) to 1.90 (item 32). The distribution of item frequency scores was positively skewed for all items, and the median frequency score was “1”. The percentage of respondents who answered “2” or above for each item, which indicated exposure to the situation, ranged from 4.3 (item 13) to 44.1 (item 1). Over 30% of the respondents answered “2” or above for three of the items (items 1, 3, and 32). For the difficulty scale, mean scores for the 40 items ranged from 1.42 (item 34) to 2.30 (item 4). The distribution of difficulty scores for all items was positively skewed, and the median difficulty score was “1” for all items except for items 1 and 3 (median = 2).

**Figure 1:**
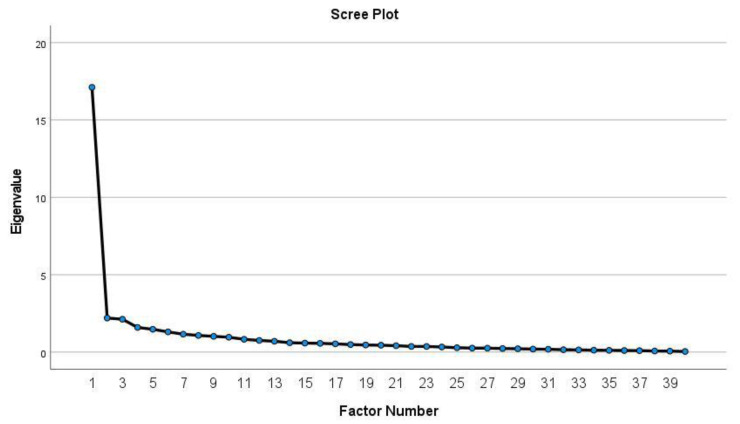
Scree Plot for Factor Analysis of Situational Items–Frequency Scale (*N* = 40).

*Exploratory Factor Analysis*. The 40 situational items of the Phase 2 survey were entered into an inter-item correlation matrix and were factor analyzed. The inter-item correlation matrix of the frequency scale indicated primarily low to moderate correlations among the 40 situational items, with many of the items ranging from 0.20 to 0.50. A scree plot of the frequency scale demonstrated a visible “dip”, followed by a flattening of the curve, that strongly suggested a 3-factor solution (See Figure 1). However, to ensure all potentially viable solutions, we examined 4- and 5-factor solutions for interpretability. Comparing all potentially viable solutions, the 3-factor solution appeared to have the most conceptually interpretable solution and accounted for 50% of the common variance. Factors 1 through 3 from the frequency scale had eigenvalues of 16.65, 1.69, and 1.64, respectively, and accounted for 24%, 16%, and 10% of the variance, respectively. Principal axis factoring with Varimax rotation (rather than Promax rotation) was used to calculate the variance explained by each factor, as an orthogonal rotation method was necessary to calculate variance specific to each factor.

A factor solution was created by examining the difference between loadings for each item on each factor, adhering to a 0.55–0.30–0.20 rule [27,28]. Table 4 lists the 23 retained items with their respective factor loadings and communality estimates. Factor 1 comprised of fifteen items that focused on offers to use e-cigarettes from friends. The friends who offered e-cigarettes in these situations appeared to be platonic in nature. Factor 2 comprised of five items that focused on offers to use e-cigarettes from non-friends. These individuals included boyfriends/girlfriends, family members, and bullies. Three of the items in this factor also focused on marijuana vaping offers. Factor 3 comprised of 3 items that focused on coercive pressure to use e-cigarettes. These items focused on manipulative or forced demands for youths to use e-cigarettes.

Subscale scores for the impact items were calculated using the mean for items within each factor. In order, the means for Subscales 1 through 3 were 1.40, 1.19, and 1.14 (*SD*s = 0.63, 0.49, and 0.35, respectively). The inter-subscale correlations derived from the 3-factor solution are reported in Table 5. Correlations among subscales ranged from 0.45 (Subscales 2 and 3) to 0.65 (Subscales 1 and 2).

### 4.2. Phase 4: Reliability

As a measure of internal consistency, Cronbach’s alpha was calculated for each of the subscales derived from the factor analysis of frequency scores. Reliability coefficients were 0.94, 0.82, and 0.57 for Subscales 1 through 3, respectively. While the coefficients for Subscales 1 and 2 were in the excellent and good ranges, respectively, the coefficient for Subscale 3 was in the poor (although acceptable) range [31].

### 4.3. Phase 5: Validity

*Hypothesis 1*. In order to examine the association of grade level with exposure to e-cigarette offer situations, a series of one-way ANOVA tests were conducted. First, the association between Subscales 1–3 and grade level were examined. There were no statistically significant differences between group means as determined by one-way ANOVAs, although Subscale 1 approached significance, *F*(2, 252) = 2.72, *p* = 0.07. Next, the association between the top 10 most frequently experienced situations (See Table 6) and grade level were examined. Three items demonstrated statistically significant differences (Items 29, 32, and 33, see Table 7). Post hoc comparisons of these items using Tukey HSD identified significant changes in the intended direction. Sixth-grade students were less exposed to Item 29 (*p* < 0.05), Item 32 (*p* < 0.01), and Item 33 (*p* < 0.05) compared to both 7th- and 8th-grade students. Further, 7th-grade students were less exposed to Item 33 compared to 8th-grade students (*p* < 0.05).

*Hypothesis 2*. Because of the correlations between the three subscales (see Table 5), a series of semi-partial correlations were conducted to examine the association between exposure to e-cigarette offer situations and e-cigarette use. Each subscale was correlated with vaping flavors/e-liquid or marijuana vaping, with the influence of the other two subscales and the other vaping behavior removed from the relationship. Two of the predicted relationships were significant. Subscale 1 was positively correlated with vaping flavors/e-liquid, *r*(248) = 0.19, *p* < 0.01, and Subscale 2 was positively correlated with marijuana vaping, *r*(248) = 0.35, *p* < 0.001. Further, Pearson’s correlations (not presented) found significant associations between each of the 10 most frequently experienced situations (see Table 5), vaping flavors/e-liquid, and marijuana vaping (all *p*s < 0.001, except for marijuana vaping and Item 1, *p* < 0.01, Item 7, *p* < 0.05, and Item 29, *p* < 0.01).

*Hypothesis 3*. Pearson’s correlations were used to assess the relationship between the top 10 most difficult e-cigarette offers (see Table 8) and e-cigarette use. For vaping flavors/e-liquid, significant positive relationships were found for Item 3, *r*(246) = 0.13, *p* < 0.05, Item 5, *r*(250) = 0.14, *p* < 0.05, and Item 40, *r*(246) = 0.15, *p* < 0.05. For marijuana vaping, significant positive relationships were found for Item 5, *r*(250) = 0.18, *p* < 0.01, and Item 40, *r*(246) = 0.13, *p* < 0.05.

## 5. Discussion

This study examined the socially and developmentally specific e-cigarette offer situations experienced by a predominantly rural NHPI sample of youths. Test development and validation procedures were used to examine the content, factor, and construct validity and reliability of the items. In Phases 1 and 2, items were generated through a series of focus groups with rural NHPI youths and were edited to eliminate redundant or non-representative items. Phase 3 addressed the factor validity of the questionnaire. Three factors emerged from the analysis of the frequency scale: (1) E-Cigarette Offers from Friends, (2) E-Cigarette Offers from Non-Friends, and (3) Coercive Pressure to Use E-Cigarettes. Communality estimates for the retained survey items were primarily in the moderate range, with approximately 75% of them in the 0.5 to 0.6 range, suggesting the items adequately reflected the proposed factor structure. While Cronbach’s alpha scores indicated high internal consistency for Subscales 1 and 2, Subscale 3 was in the poor (although acceptable) range. Further, two of the three items within Subscale 3 had relatively low communality estimates, suggesting potential measurement issues for this subscale. Three hypotheses were tested to establish the construct validity of the items. The findings from these tests largely validated the ability of the subscales and items to assess e-cigarette use for youths in rural Hawai‘i.

The situational survey items were developed using a culturally grounded approach, in which youths’ narratives were the foundation for the items, rather than a theoretical approach, which guides the generation of items across proposed or predetermined conceptual domains. This often results in an imbalance of potential items across factors, which could have been the cause of the instability of Factor 3 in this study. However, supporting the validity of Factor 3, alternative factor analytic strategies, such as varying the rotational method, were explored in this study, which resulted in similar 3-factor solutions. Future research with rural and/or NHPI youth populations might consider adding additional items reflecting coercive pressure to use e-cigarettes, ideally with a larger sample, to clearly establish this factor.

The findings highlight the ecodevelopmental contributors to e-cigarette use for rural NHPI youths. The situations in the survey reflected that e-cigarettes are offered primarily in the school setting but also in the home and community settings. Further, the factor structure highlighted the relational contributors to e-cigarette offers within rural ecodevelopmental contexts, as it was primarily characterized by types of offerors (i.e., friends versus non-friends). These findings are consistent with prior test development research in rural Hawai‘i, which found a similar factor structure organized by types of substance use offerors [21] and highlights the salience of the social and relational contexts of substance use for NHPI and Indigenous youth populations [7,32,33]. NHPI culture is highly communal, with a high degree of interdependence and reliance among family and community members [34]. This communal nature is particularly pronounced for NHPIs in rural areas [35]. The present study findings are reflective of these sociocultural norms and characteristics. They suggest that e-cigarette offers from friends are received differently by rural NHPI youths than those from family members and that different strategies may need to be employed based on the type of offeror in each situation for youths to maintain relational harmony in rural communities on Hawai‘i Island. For example, saying “no” to an e-cigarette offer might be socially and culturally acceptable for these youths when the offeror is a peer or friend but not when the offeror is a parent or cousin. The latter pair of offerors may consider the abruptness of saying “no” to an e-cigarette offer as disrespectful. 

### 5.1. Implications for Practice

This study has implications for culturally specific practice with rural Indigenous and NHPI youths. The survey can be used to assess the ecodevelopmental risk and protective factors for these youths’ e-cigarette use. Further, since increased exposure to offers to use e-cigarettes was strongly associated with e-cigarette use in the present study, the need for tailored interventions to reduce these offers for youths in rural Hawai‘i is indicated. Recent research has found that prior strategies used by youths to reduce substance use offers, such as overt refusals, explanations, avoidance, and leaving, were also used to reduce offers to use e-cigarettes, but the widespread and pervasive nature of electronic devices in rural Hawai‘i may have decreased the overall efficacy of these strategies [13]. Interventions might use the situations and subscales in this study as a foundation for culturally grounded resistance skills training in the context of widespread e-cigarette use in rural and/or NHPI homes, schools, and communities. This may require increased emphasis on assertiveness in using resistance skills within the situations described in this study, as well as effective methods to combine different resistance skills, such as using overt refusal with explanations or avoidance, to address the situations in this study. Ultimately, the goal would be to limit youths’ exposure to e-cigarette offers across multiple ecodevelopmental contexts. 

### 5.2. Limitations of the Study

This study had several limitations. Because active parental consent was required for youths’ participation in this study, the sample may have been influenced by a selection bias. Past research has indicated that students who are the most at risk for drug use are often not given parental consent to participate in studies in rural Hawai‘i [7,21]. This selection bias may have influenced the generalizability of the findings to the community at large, as well as to other rural communities with concentrations of NHPI youths across the state. Further, because of the rural setting of the study, findings may not be generalizable to urban communities in Hawai‘i. Additionally, the relationship of the survey to social desirability was not assessed. The sensitive nature of the survey topic may have caused respondents to deny or overstate their exposure to e-cigarettes, potentially affecting the validity of the findings.

## 6. Conclusions

Nearly one in five middle school youths in Hawai‘i currently use an electronic vapor product, with NHPI youths representing the highest number of active e-cigarette users among the major ethnic groups in the state [36]. Compared to the state of Hawai‘i, even higher rates of e-cigarette use exist for middle school youths across the United States Affiliated Pacific Island territories, such as Guam and the Marshall Islands [36]. This study addresses the high rates of youth e-cigarette use across the Pacific region by identifying culturally and developmentally specific e-cigarette offer situations for NHPI youths and analyzing these situations using test development and validation procedures. The study findings have direct implications for the assessment and prevention of e-cigarette use for rural, NHPI, and/or Indigenous youth populations. The survey can be used to assess ecodevelopmental risk and protective factors across different youth contexts, and the situational items in the survey could be used to practice resistance skills and promote critical thinking within problematic situations involving e-cigarettes. Future research should continue to refine the psychometric properties of the survey, particularly in the area of coercive pressure to use e-cigarettes, with additional samples across different Hawaiian Islands and/or across the Pacific region and with additional related substudies that provide further validation for the survey. A future, refined measure could then be evaluated for goodness of fit using confirmatory factor analytic procedures with a unique sample of NHPI youths.

## Figures and Tables

**Table 1:** Selected Questions from the Phase 1 Semi-Structured Interview Schedule.

1.	Have you or someone you know ever been offered e-cigarettes, something similar to e-cigarettes (like vape pens), or tobacco cigarettes? If so, what did you/they do?
2.	Where do kids use e-cigarettes, similar devices, or tobacco cigarettes on Hawai‘i Island?
3.	Have you or someone you know been invited to go with kids who planned to use e-cigarettes, similar devices, or tobacco cigarettes? What did you/they do?

**Table 2:** E-Cigarette Survey Items: Item Refinement and Selection.

Your classmate has pulled the sleeve of his hoodie over his hand to hide his vape pen and is vaping in class through his sleeve. He looks up at you and says, “Eh, you like try?”
2. A big kid follows you and your friend into the bathroom at recess. He corners you and tries to make you buy a vape.
3. Your close friend, whom you have known for a long time, keeps asking you over and over if you want to try vaping with him/her.
4. Someone tells you that he/she is going to spread a nasty rumor about you unless you try vaping with him/her.
5. During a family gathering, your older cousins are going to vape around the side of your uncle’s house. They ask you if you want to join them.
6. You go to use the restroom at recess and run into someone who is high from using a marijuana dab pen. (S)he asks you if you want to take a hit.
7. Your friend asks you to try vaping because he/she does not want to vape alone.
8. Kids are passing around a vape beneath the desks in class. When it gets to you, the student who passes it to you says, “Eh, go smoke ‘um at recess.”
9. You run into two friends vaping inside the shopping mall. They ask you if you want to take a hit.
10. You are at the gym for PE, and everyone is doing different activities. Your friend asks you if you want to go behind the bleachers to vape.
11. Your favorite cousin asks you if you want to try vaping. You are afraid of what (s)he will think of you if you say no.
12. You are in the park, and a couple of kids from your school approach you near the bathroom. They ask you if you want to try their vape.
13. Your mom or dad offers to buy you a vape.
14. You walk into the bathroom at school and smell perfume/cologne. You see two classmates coming out of the bathroom stall, and they ask if you want to try vaping.
15. Your brother/sister and their friend are getting high from a marijuana dab pen in your brother/sister’s bedroom. You walk in on them, and they ask you if you want to try it.
16. A group of kids ask if you want to try vaping. You are afraid they’ll think you are lame if you say no.
17. A group of kids is on Instagram watching someone from their school do tricks with their vape. They ask you if you want to learn how to do them.
18. Your older brother/sister leaves his/her vape in their room. They will not notice if you take hits off of it when they are not home.
19. You are at a sleepover at a friend’s house, and someone offers you to take a hit off of a marijuana dab pen.
20. There are a couple of kids at school who sell vape and have asked you more than once if you’d like to buy one.
21. Someone you know at school is using a marijuana dab pen and offers to sell you a hit from it.
22. You are sitting at the back of the bus, and the kids across from you are vaping. They look over at you and offer to let you vape with them.
23. A classmate is using a mod and blows the smoke into their backpack to hide it. (S)he gives you a look that makes you think they want you to try it.
24. You are sitting in a parked car with friends who are vaping, and the vape is passed to you.
25. Kids who vape go to a part of the school that is off-limits because they know teachers will not monitor that area. Your friends start walking over there and tell you to come along.
26. Your friend tells you that the smoke from vaping is not as harmful if you just puff on it rather than take a big inhale, so you should try it.
27. Your friend keeps pestering you to try vaping and tells you that it is good for you
28. You are feeling really sad, and your friend says taking a hit from their marijuana dab pen will help you feel better.
29. During class, kids take a hit from a vape device and blow the smoke into their shirts. They tell you to take a hit and do the same thing.
30. Your boyfriend or girlfriend offers you their vaping device to use.
31. Your friend offers you a hit off his/her vape and tells you that you will not get addicted.
32. Older family members (parents, aunties, uncles) vape in front of you and leave their vape in places where you could easily take it without them noticing.
33. Your friend has the latest vape flavor and offers to let you try it.
34. There are places that are dark where vaping devices cannot be seen (e.g., movie theatres). While inside one of these places, your friend asks you to take a hit off of his/her vape.
35. You and your friends think the older kids in your school look cool when they vape. Your friend asks you if you want to try it.
36. Someone tells you to try out their marijuana dab pen for something stronger than regular vaping.
37. Everyone around you is vaping, and you are the only one who is not. Your friend offers you a hit.
38. Your older sibling has a marijuana dab pen. It would be easy to steal it from them and use it.
39. A bigger kid in your school offers you a vape and says (s)he’ll beat you up if you say no.
40. Your friend is going to have a bag search. (S)he asks you to hide their vape device and says you can use it.

**Table 3:** Participant Demographics (*N* = 257).

Variable		*M*	*SD*	%
Gender				
	Male			49.8
	Female			49.0
	Non-Conforming			<1
Age		12.71	0.87	
Grade				
	7th			59.2
	8th			23.1
	6th			17.6
Ethnicity *				
	Hawaiian/Part Hawaiian			60.7
	Filipino			47.9
	White			35.4
	Portuguese			31.5
	Chinese			29.2
	Japanese			28.0
	Other			25.3
	Hispanic/Latino/Spanish			22.2
	Other Pacific Islander			11.3
	African American			8.9
	Samoan			7.8
	Other Asian			7.4
	Korean			3.1
Free/Reduced Cost Lunch				85.2

* Participants were allowed to select more than one ethnicity; the percentages do not equal 100%.

**Table 4:** Factor Loadings and Communality Estimates (h^2^) for Retained Items using Principal Axis Factoring and Promax Rotation.

Item		Factor 1	Factor 2	Factor 3	h^2^
37.	Everyone around you is vaping, and you are the only one who is not. Your friend offers you a hit.	**0.941**	−0.145	0.020	0.702
34.	There are places that are dark where vaping devices cannot be seen (e.g., movie theatres). While inside one of these places, your friend asks you to take a hit off of his/her vape.	**0.826**	0.151	−0.257	0.691
33.	Your friend has the latest vape flavor and offers to let you try it.	**0.797**	0.096	−0.068	0.693
27.	Your friend keeps pestering you to try vaping and tells you that it is good for you.	**0.782**	−0.242	0.075	0.460
31.	Your friend offers you a hit off his/her vape and tells you that you will not get addicted.	**0.726**	0.084	0.035	0.648
9.	You run into two friends vaping inside the shopping mall. They ask you if you want to take a hit.	**0.712**	0.219	−0.267	0.594
1.	Your classmate has pulled the sleeve of his hoodie over his hand to hide his vape pen and is vaping in class through his sleeve. He looks up at you and says, “Eh, you like try?”	**0.684**	−0.236	0.284	0.526
40.	Your friend is going to have a bag search. (S)he asks you to hold and hide their vape device and says you can use it.	**0.681**	0.101	0.010	0.578
35.	You and your friends think the older kids in your school look cool when they vape. Your friend asks you if you want to try it.	**0.671**	−0.128	0.259	0.568
7.	Your friend asks you to try vaping because he/she does not want to vape alone.	**0.645**	−0.261	−0.328	0.507
3.	Your close friend, whom you have known for a long time, keeps asking you over and over if you want to try vaping with him/her.	**0.643**	0.005	0.107	0.502
26	Your friend tells you that the smoke from vaping is not as harmful if you just puff on it rather than take a big inhale, so you should try it.	**0.623**	0.013	0.206	0.578
25.	Kids who vape go to a part of the school that is off-limits because they know teachers will not monitor that area. Your friends start walking over there and tell you to come along.	**0.615**	0.085	0.161	0.599
29.	During class, kids take a hit from a vape device and blow the smoke into their shirts. They tell you to take a hit and do the same thing.	**0.587**	0.176	0.105	0.610
28.	You are feeling really sad, and your friend says taking a hit from their marijuana dab pen will help you feel better.	**0.553**	0.246	−0.008	0.548
30.	Your boyfriend or girlfriend offers you their vaping device to use.	0.105	**0.788**	−0.112	0.671
15.	Your brother/sister and their friend are getting high from a marijuana dab pen in your brother/sister’s bedroom. You walk in on them, and they ask you if you want to try it.	−0.227	**0.784**	0.150	0.507
39.	A bigger kid in your school offers you a vape and says (s)he’ll beat you up if you say no.	−0.154	**0.675**	0.247	0.499
21.	Someone you know at school is using a marijuana dab pen and offers to sell you a hit from it.	0.057	**0.607**	0.179	0.555
38.	Your older sibling has a marijuana dab pen. It would be easy to steal it from them and use it.	0.085	**0.554**	−0.079	0.342
8.	Kids are passing around a vape beneath the desks in class. When it gets to you, the student who passes it to you says, “Eh, go smoke ‘um at recess.”	−0.077	0.278	**0.636**	0.557
4.	Someone tells you that he/she is going to spread a nasty rumor about you unless you try vaping with him/her.	−0.054	0.038	**0.600**	0.347
2.	A big kid follows you and your friend into the bathroom at recess. He corners you and tries to make you buy a vape.	−0.028	−0.071	**0.564**	0.277

**Table 5:** Inter-Subscale Correlations Derived from the Three-Factor Solution.

	Subscale 1	Subscale 2	Subscale 3
Subscale 1	1.00		
Subscale 2	0.65	1.00	
Subscale 3	0.50	0.45	1.00

Note: Subscale 1 = E-Cigarette Offers from Friends; Subscale 2 = E-Cigarette Offers from Non-Friends; Subscale 3 = Coercive Pressure to Use E-Cigarettes.

**Table 6:** Most Frequently Experienced E-Cigarette Offer Situations (*n* = 10).

Item		*N*	*M*	*SD*
32.	Older family members (parents, aunties, uncles) vape in front of you and leave their vape in places where you could easily take it without them noticing.	252	1.90	1.43
1.	Your classmate has pulled the sleeve of his hoodie over his hand to hide his vape pen and is vaping in class through his sleeve. He looks up at you and says, “Eh, you like try?”	256	1.84	1.14
3.	Your close friend, whom you have known for a long time, keeps asking you over and over if you want to try vaping with him/her.	253	1.55	0.94
37.	Everyone around you is vaping, and you are the only one who is not. Your friend offers you a hit.	254	1.52	0.98
40.	Your friend is going to have a bag search. (S)he asks you to hide their vape device and says you can use it.	255	1.44	0.93
20.	There are a couple of kids at school who sell vape and have asked you more than once if you’d like to buy one.	253	1.40	0.80
5.	During a family gathering, your older cousins are going to vape around the side of your uncle’s house. They ask you if you want to join them.	254	1.40	0.93
33.	Your friend has the latest vape flavor and offers to let you try it.	253	1.40	0.89
7.	Your friend asks you to try vaping because he/she does not want to vape alone.	255	1.39	0.87
29.	During class, kids take a hit from a vape device and blow the smoke into their shirts. They tell you to take a hit and do the same thing.	254	1.36	0.84

**Table 7:** Means, Standard Deviations, and One-Way Analyses of Variance for E-Cigarette Situations–Frequency Scale.

Item	6th Grade		7th Grade		8th Grade		*F*(2, 247–249)	η^2^
	M	SD	M	SD	M	SD		
29.	1.07	0.33	1.41	0.91	1.48	0.87	3.70 *	0.03
32.	1.44	0.96	1.89	1.46	2.33	1.55	4.97 **	0.04
33.	1.23	0.60	1.34	0.80	1.70	1.19	4.56 *	0.04

* *p* < 0.05, ** *p* < 0.01.

**Table 8:** Most Difficult E-Cigarette Offer Situations (*n* = 10).

Item		*N*	*M*	*SD*
4.	Someone tells you that he/she is going to spread a nasty rumor about you unless you try vaping with him/her.	252	2.30	1.39
2.	A big kid follows you and your friend into the bathroom at recess. He corners you and tries to make you buy a vape.	254	2.02	1.17
39.	A bigger kid in your school offers you a vape and says (s)he will beat you up if you say no.	250	2.02	1.35
40.	Your friend is going to have a bag search. (S)he asks you to hide their vape device and says you can use it.	254	1.82	1.16
11.	Your favorite cousin asks you if you want to try vaping. You are afraid of what (s)he will think of you if you say no.	251	1.79	1.06
3.	Your close friend, whom you have known for a long time, keeps asking you over and over if you want to try vaping with him/her.	250	1.79	1.05
16.	A group of kids ask if you want to try vaping. You are afraid they’ll think you are lame if you say no.	250	1.66	0.94
5.	During a family gathering, your older cousins are going to vape around the side of your uncle’s house. They ask you if you want to join them.	254	1.64	0.96
7.	Your friend asks you to try vaping because he/she does not want to vape alone.	252	1.63	0.93
28.	You are feeling really sad, and your friend says taking a hit from their marijuana dab pen will help you feel better.	246	1.62	1.07

## Data Availability

Deidentified raw data supporting the conclusions of this article will be made available by the authors on request.
